# The Potential Role of REG Family Proteins in Inflammatory and Inflammation-Associated Diseases of the Gastrointestinal Tract

**DOI:** 10.3390/ijms22137196

**Published:** 2021-07-03

**Authors:** Chao Sun, Xiaoyu Wang, Yangyang Hui, Hirokazu Fukui, Bangmao Wang, Hiroto Miwa

**Affiliations:** 1 Department of Gastroenterology and Hepatology, Tianjin Medical University General Hospital, Anshan Road 154, Heping District, Tianjin 300052, China; chaosun@tmu.edu.cn (C.S.); wxy_judy@tmu.edu.cn (X.W.); yyhui@tmu.edu.cn (Y.H.); mwang02@tmu.edu.cn (B.W.); 2 Tianjin Institute of Digestive Disease, Tianjin Medical University General Hospital, Anshan Road 154, Heping District, Tianjin 300052, China; 3 Division of Gastroenterology and Hepatology, Department of Internal Medicine, Hyogo College of Medicine, Nishinomiya 663-8501, Japan; miwahgi@hyo-med.ac.jp

**Keywords:** REG family proteins, inflammation, inflammatory bowel disease, gastric cancer, colorectal cancer, mitogenesis

## Abstract

Regenerating gene (REG) family proteins serve as multifunctional secretory molecules with trophic, antiapoptotic, anti-inflammatory, antimicrobial and probably immuno-regulatory effects. Since their discovery, accumulating evidence has clarified the potential roles of the REG family in the occurrence, progression and development of a wide range of inflammatory and inflammation-associated diseases of the gastrointestinal (GI) tract. However, significant gaps still exist due to the undefined nature of certain receptors, regulatory signaling pathways and possible interactions among distinct Reg members. In this narrative review, we first describe the structural features, distribution pattern and purported regulatory mechanisms of REG family proteins. Furthermore, we summarize the established and proposed roles of REG proteins in the pathogenesis of various inflammation-associated pathologies of the GI tract and the body as a whole, focusing particularly on carcinogenesis in the ulcerative colitis—colitic cancer sequence and gastric cancer. Finally, the clinical relevance of REG products in the context of diagnosis, treatment and prognostication are also discussed in detail. The current evidence suggests a need to better understanding the versatile roles of Reg family proteins in the pathogenesis of inflammatory-associated diseases, and their broadened future usage as therapeutic targets and prognostic biomarkers is anticipated.

## 1. Introduction

Regenerating gene (Reg) family proteins are divided into four subgroups (Reg I, Reg II, Reg III and Reg IV) on the basis of their primary structure. The expression patterns and pathophysiological roles of these proteins have been extensively investigated in rodents and humans. In rodents, Reg family proteins comprise Reg I, Reg II, Reg IIIα, Reg IIIβ, Reg IIIγ, Reg IIIδ and Reg IV, while those in human are REG Iα, REG Iβ, REG IIIα, REG IIIγ and REG IV [1]. REG proteins contain a C-type-like lectin domain, which facilitates selective binding to a variety of carbohydrates and ligands [2]. The binding interaction is mediated by the expression of specific anionic residues as well as in a Ca^2+^-independent manner. For instance, REG IV, pertaining to human beings, represents binding affinity to mannan or heparin via two Ca^2+^-independent sites, while REG III recognizes peptidoglycan carbohydrate moiety through a tripeptide motif [3,4]. Another structural feature of REG proteins is characterized by a cleavage site for trypsin in the vicinity of the N-terminus [5]. A soluble short peptide and the remaining insoluble fragment consisting of approximate 130 residues are generated in response to trypsin digestion [6]. The latter is activated upon cleavage of a peptide sequence without definite physiological role. Given the fact that the various REG genes show approximately 50–70% homology, they appear to function similarly in terms of mitogenic and antiapoptotic activity and also differentiation induction in a wide range of diseases and physiological conditions. We and others have shown that REG proteins are involved in the pathogenesis of several conditions driven by inflammation in the gastrointestinal (GI) tract and body as a whole, including inflammatory bowel disease (IBD), indomethacin-induced GI injury and rheumatoid arthritis (RA) [7,8,9]. Additionally, REG family proteins can be used for prognostication of digestive tract malignancies, thus serving as potential diagnostic markers or therapeutic targets [10].

In the present review, we provide an overview of REG family proteins, including their regulatory signaling pathways and possible functions, and discuss their potential roles in the occurrence and development of inflammatory diseases (predominantly those of the GI tract), inflammation-associated carcinogenesis and their clinical application as biomarkers of inflammatory/neoplastic pathologies.

## 2. Background of the Reg Family

The first Reg gene was discovered by Terazono et al. in 1988 and was shown to contribute to β-cell regeneration in vitro and in vivo [11,12]. REG genes each have six exons and five introns, with the exception of REG IV, which has seven exons. In humans, REG IV gene is located on chromosome 1p11-3, whereas the others are on chromosome 2p12 [13]. REG family proteins have a molecular mass of 16-17 kDa. Reg I is present in pancreatic acinar cells, which are capable of transdifferentiation to islets in pathological states but not under healthy conditions [14,15]. Recently, it has been shown that Reg I is expressed in gastric enterochromaffin-like (ECL) cells and that its production is stimulated by gastrin, acting as a mitogenic factor to enhance the proliferation of gastric epithelial cells [16,17]. Reg II is detected only in mice and has no human ortholog, and *Reg II* mRNA is known to be expressed predominantly in normal pancreatic acini and hyperplastic islets [18]. The third subgroup of Reg proteins is expressed mainly in the pancreas, acting to ameliorate stress and prevent pancreatic inflammation. In this context, Reg IIIβ may be expressed in response to nuclear factor kappa B (NF-κB) and tumor necrosis factor-α (TNF-α), subsequently exerting antiapoptotic and anti-inflammatory actions during oxidative stress [19,20,21]. On the other hand, IL-22 signaling can induce intestinal expression of REG IIIα and REG IIIγ to enhance the survival of intestinal stem cells and Paneth cells [22,23]. The most recently discovered member, REG IV, was originally isolated by Hartupee et al. from an ulcerative colitis (UC) cDNA library [24]. REG IV is expressed in various human organs and tissues, such as the colon, small intestine, stomach, pancreas and appendix [25,26].

## 3. Regulation of Reg Family Genes

**Figure 1:**
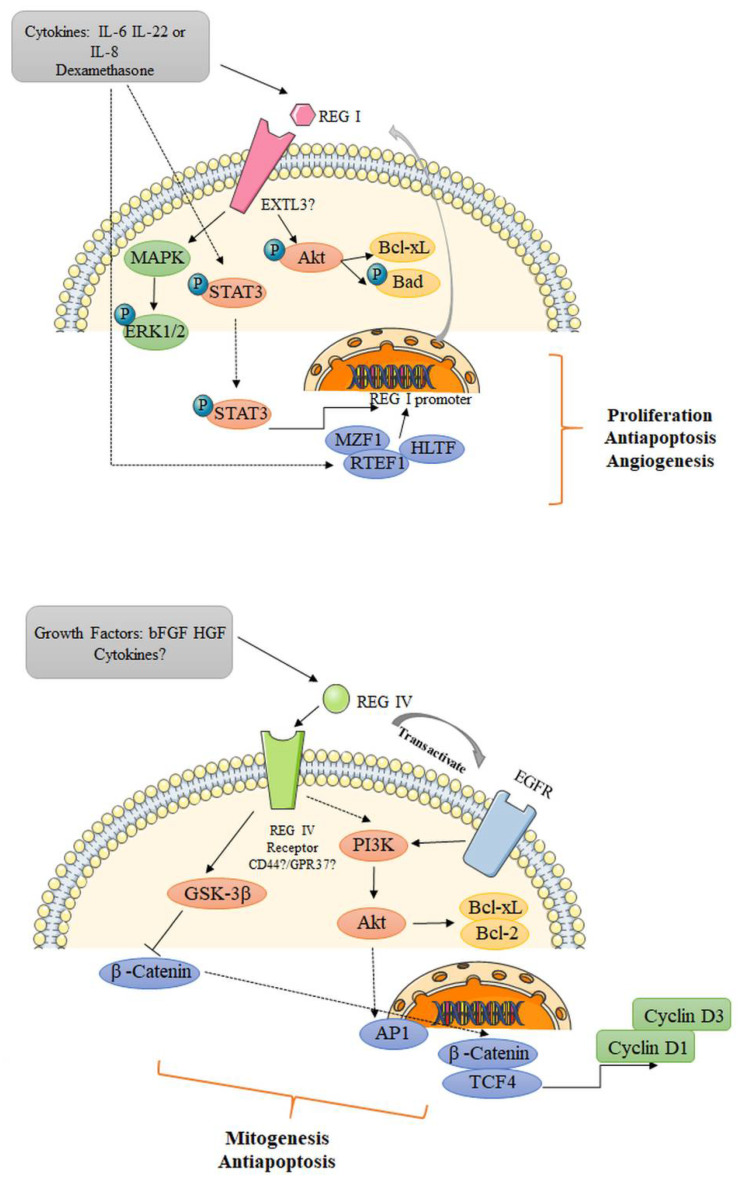
Potential mechanism of REG I and REG IV signaling. Upon stimulation from exogenous growth factors or cytokines, REG I/REG IV bind to their putative receptors and subsequently activate or transactivate several signaling cascades, serving as mitogenic, antiapoptotic and/or angiogenic factors in the pathogenesis of various pathological conditions. EXTL3, exostoses-like gene 3; MAPK, mitogen-activated protein kinase; ERK, extracellular signal-regulated kinase; STAT3, signal transducer and activator of transcription 3; MZF1, myeloid zinc finger 1; RTEF1, related transcriptional enhancer factor-1; HLTF, helicase-like transcription factor; bFGF, basic fibroblast growth factor; HGF, hepatocyte growth factor; AP1, activator protein-1; EGFR, epidermal growth factor receptor; GSK-3β, glycogen synthase kinase 3β; TCF4, T-cell factor 4.

The specific receptors of Reg remain elusive, and this has hindered classification of the molecular pathways in which these proteins function. Kobayashi et al. identified a cDNA for the rat Reg I protein receptor based on an islet cDNA expression library using a ^125^I-labeled protein probe [27]. This receptor corresponded to human multiple exostoses-like gene 3 (EXTL3), which contributes to the biosynthesis of heparan sulfate (Figure 1). The *EXTL3* mRNA is broadly present in GI tract epithelia, as well as the pancreas, liver, spleen, kidney, neurons and various glands. Further studies have revealed that overexpression of EXTL3 in β-cells triggers the expression of activating transcription factor-2 (ATF-2) [28], and that REG Iα regulates neurite outgrowth via its receptor EXTL3 in neuronal cell lines and primary hippocampal neurons [29]. These findings suggest the possible involvement of the REG I–REG receptor signaling system in a wide range of cell types. On the other hand, it has been speculated that CD44 may be a REG IV receptor, as the two show interaction in colorectal cancer (CRC) proliferation [30]. Intriguingly, Wang et al. have shown that G protein-coupled receptor 37 (GPR37) is part of the same complex as REG IV, which mediates its signal transduction and promotes peritoneal metastasis of gastric cancer cells [31].

There is now mounting evidence that exogenous growth factors and several cytokines induce *REG* gene expression (Figure 1). Akiyama et al. have reported that a combination of IL-6 and dexamethasone enhanced the expression of *Reg I* mRNA in the rat insulinoma cell line RINm5F. This effect was attributable to the presence of a cis-element of the *Reg* gene promoter (T^−81^GCCCCTCCCAT^−70^) and poly(ADP-ribose) synthetase/polymerase inhibitors promoting Reg production and secretion at the transcriptional level [32]. Dusetti et al. have also shown that IL-6 and dexamethasone together strongly upregulate Reg I in acinar AR-42J cells, whereas this positive effect is partly eliminated by addition of IL-1 [33]. It has been reported in ECL cells, a cytokine-induced neutrophil chemoattractant, dose-dependently increased the expression of *Reg I* mRNA and protein via CXC receptor 2 during the healing of gastric mucosal damage [34]. In *H. pylori*-infected human gastric mucosa, IL-8 might stimulate REG protein production [35]. In addition, both IL-22 and IL-10, which share IL-10 receptor β, can upregulate Reg II by activating the signal transducer and activator of transcription 3 (STAT3) in acinar cells [36,37]. Sekikawa et al. have shown that addition of IL-6 promotes the expression of REG Iα protein via the STAT3 pathway [38]. The *REG Iα* promoter region is located in the sequence from −142 to −134 as an IL-6 responsive element, and enhanced REG Iα activates Akt, p-Bad and Bcl-xL as an antiapoptotic factor in gastric cancer cells. A REG IIIα-JAK2/STAT3 positive loop has been suggested to promote tumor formation in human pancreatic cancer, which is mediated by EGFR [39]. Furthermore, Naito et al., using a reporter gene assay, have revealed that transcriptional caudal type homeobox 2 (CDX2) DNA-binding elements are located in the 5’-flanking region of the *REG IV* gene [40]. The ability of CDX2 to bind directly to REG IV has been further verified by chromatin immunoprecipitation assays.

## 4. Reg and Inflammatory Diseases (GI Tract and Systemic Inflammation)

IBD is an inflammatory GI tract disease affecting millions of patients worldwide. The putative etiology includes excessive exposure of the intestine to microbiota, aberrant activation of the immune system, impaired epithelial barrier function and subsequent injury of intestinal tissues. Two distinct IBD disorders have been identified: Crohn’s disease (CD) and UC. UC and CD each have a discrete cytokine profile. In UC, T-helper type 2 (Th2) cytokine IL-5/IL-13 are the main players [41], whereas in CD, the Th1 cytokine interferon-γ and the Th17 cytokines IL-17/IL-22 are actively involved. The possible mechanisms, pathogenetic involvement and clinical relevance of REG proteins in IBD have recently been outlined by others [1], and we will mention them only briefly here. In a dextran sulfate sodium (DSS)-induced mouse model of colitis, Ogawa et al. found that epithelial Reg IIIβ and Reg IIIγ were overexpressed, possibly indicating mucosal inflammation driven by commensal bacteria [42]. In the same disease model, Xu et al. demonstrated that Reg IIIβ and Reg IIIγ were alternatively increased in the colonic epithelia, their expression levels being correlated with those of STAT3-associated cytokines such as IL-6, IL-17 and IL-22 [43]. In LoVo and SW403 colon cancer cells, IL-22 stimulation has been suggested to enhance the expression of REG Iα by activating its gene promoter through the STAT3 pathway [7]. Another study has revealed that REG IIIα dose dependently repressed the production of proinflammatory cytokines and adhesive molecules in intestinal mucosa collected from subjects with active CD [42]. Intriguingly, intrarectal administration of Reg IIIα or transgenic overexpression of Reg IIIα both mitigate colonic inflammation and damage, probably through reduction of oxidative stress by preserving the gut microbiota [44]. It has also been shown that DSS-induced colitis is aggravated in Reg IIIβ^−/−^ mice relative to wild-type mice, suggesting that attenuation of colitis and ileitis is dependent on the function of Reg IIIβ [45]. Bishnupuri et al. showed that REG IV protein activates the epidermal growth factor/Akt/ activator protein-1 signaling pathway in vitro [30]. The same research group also identified REG IV as a potent regulator of mitotic division of CRC cells in humans [46]. These findings suggest that the REG IV-mediated increase in nuclear β-catenin might elicit TCF-4 transcriptional activities to promote the expression of cell cycle-regulatory genes (cyclin D1 and D3) and associated cyclin-dependent kinases. Another study has suggested that stimulation of SW430 cells with basic fibroblast growth factor and hepatocyte growth factor enhances the expression of *REG IV* mRNA via the mitogen-activated protein kinase (MAPK)-dependent pathway [47]. An increased number of studies have analyzed the expression of REG family proteins in IBD mucosa separately or in combination [1]. More recently, Takasawa and colleagues have comprehensively examined the expression of all five *REG* family mRNAs in biopsy samples harvested from IBD patients using quantitative RT-PCR [48,49]. Their results suggested that *REG Iα*, *REG Iβ* and *REG IV* mRNAs are overexpressed in the CD colon, whereas the *REG IV* gene is overexpressed in UC samples. The IL-22/IL-6-induced upregulation of *REG Iα* and *REG Iβ* is attributed to several transcription factors including myeloid zinc finger 1, related transcriptional enhancer factor-1/ TEA domain transcription factor 4, STAT3 and helicase-like transcription factor/forkhead box protein N2 in the LS-174T and HT-29 cell lines [49]. On the other hand, GATA DNA-binding protein 6 (GATA6) is pivotal for REG IV expression in colon epithelial cells under TNF-α suppression.

*Helicobacter pylori* has been identified as a critical pathogen in the development of chronic gastritis, gastric ulcer and gastric cancer. Fukui et al. have reported that the Reg gene is associated with hypergastrinemia and fundic mucosal inflammation and might participate in *H. pylori*-induced gastritis [50]. In a study of indomethacin-induced lesions, Sun et al. suggested that Reg I might play a pivotal role in maintenance of GI mucosal integrity by enhancing cell proliferation and conferring resistance to the impact of apoptosis [8]. Intriguingly, in that study, the expression of Reg IIIβ and Reg IIIγ was also significantly increased in the upper GI tract during mucosal damage, indicating that Reg family proteins might exert trophic, antiapoptotic and antimicrobial effects. In vitro data suggest that REG Iα protein might contribute to the maintenance of mucosal barrier function by inducing tight junction proteins such as claudin 3 and 4 [51]. Amebic colitis due to *Entamoeba histolytica* infection is characterized by intestinal mucosal ulcers. Peterson et al. showed that the expression of REG Iα and REG Iβ is elevated during amebiasis and thus may function to prevent parasite-induced apoptosis [52].

Reg III proteins appear to have multiple functions in the intestine. Ogawa et al. have found that epithelial expression of Reg III is upregulated under conditions of mucosal inflammation initiated by exposure to commensal bacteria [42]. Another study has shown that Reg IIIβ binds to bacterial pathogens and might interfere with their mode of action [53]. This preventive activity against intestinal translocation is observed with respect to the gram-negative bacterium *S. enteritidis* but not the gram-positive bacterium L. monocytogenes. Zheng et al. have reported that exogenous mouse or human Reg IIIγ markedly improves the survival of IL-22-knockout mice after infection with *C. rodentium* [54]. In contrast to Reg III, Reg IV is produced predominantly by deep crypt secretory cells and intestinal enteroendocrine cells in the colon [55]. Gut Reg IV- and complement factor D-mediated membrane attack complexes may help to maintain gut homeostasis by elimination of inflammatory *E. coli* [56].

**Figure 2:**
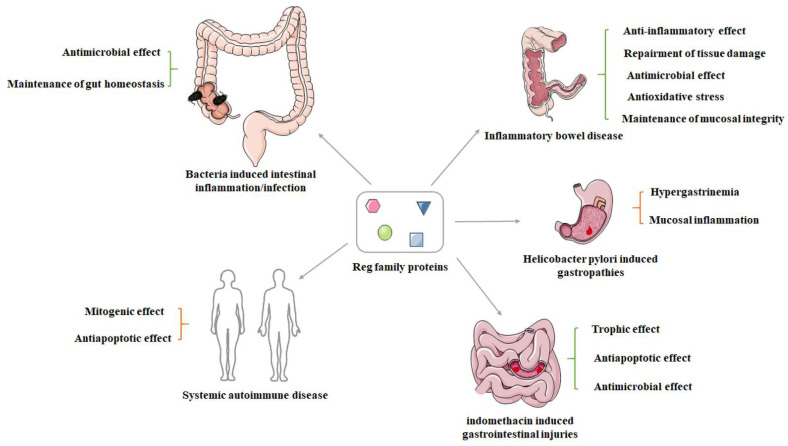
The potential role of Reg family proteins in the onset, development and progression of versatile inflammatory diseases. Mounting evidence has suggested that the Reg family proteins may serve as both protective and detrimental pathophysiological factors in the context of distinct conditions.

In addition to inflammatory response in the GI tract, REG family proteins are also involved in the pathogenesis of several systemic autoimmune diseases. Fujishiro et al. showed that REG Iα plays a significant role in the pathogenesis of RA through abnormal activation of synovial fibroblasts, subsequently giving rise to pannus formation [57]. Sjögren’s syndrome is a chronic autoimmune disease without a clear etiology, characterized by lymphocytic infiltration of salivary and lacrimal glands. Fukui et al. have suggested that REG Iα might play a role in the regeneration of ductal epithelial cells in the minor salivary glands [58]. Another study by Yoshimoto et al. has confirmed the expression of *REG Iα* mRNA in the salivary glands and REG Iα in the ductal epithelial cells of minor salivary glands from patients with primary Sjögren’s syndrome [59]. One detailed study has clarified that IL-6 stimulation triggers REG Iα transcription by activating STAT3 and its binding to the *Reg Iα* promoter in salivary ductal cells, which might be responsible for the progression of Sjögren’s syndrome [60]. A summary of these contents is illustrated in Figure 2.

## 5. Involvement of Reg Family Proteins in Inflammation-Associated Carcinogenesis

It has been widely accepted that inflammation contributes to the onset and progression of many of malignancies. In fact, patients with longstanding and extensive IBD are at increased risk of CRC [61,62]. In UC, this malignant transformation is believed to result from a chronic inflammation–dysplasia–carcinoma sequence [63]. Current practice is to perform surveillance examinations every 1-3 years for detection and removal of lesions. In the development of UC-associated neoplasia, Tanaka et al. have shown that the pattern of REG Iα immunostaining is significantly correlated with p53 overexpression [64]. Sekikawa et al. have demonstrated that REG Iα is expressed not only in the epithelia of UC mucosa but also in precancerous dysplastic epithelial cells and seven colon cancer cell lines [65]. The growth-promoting and antiapoptotic properties of REG Iα might be responsible for its role in the UC–colitic cancer sequence. REG IV, as a multifunctional secreted protein, acts on intestinal cell proliferation, migration and invasion in an autocrine and paracrine manner in HT-29 cell lines [66]. Zhang et al. have demonstrated that REG IV expression is positively correlated with severity of dysplasia in adenoma, suggesting that overexpression of REG IV might be an early event in colorectal carcinogenesis [67].

**Figure 3:**
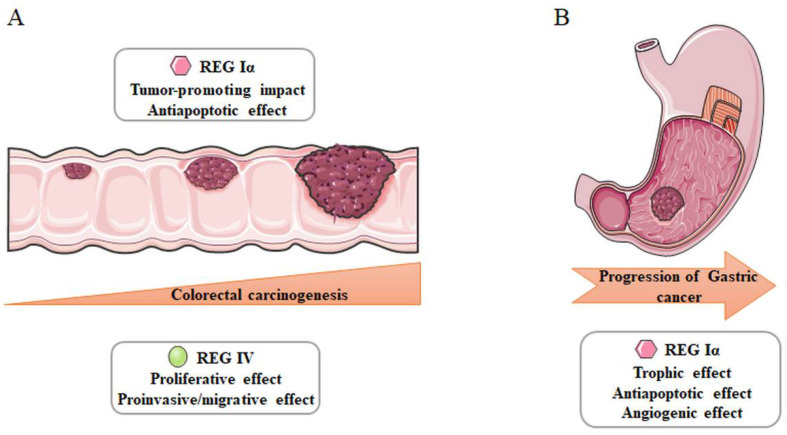
Involvement of REG family proteins in inflammation-associated carcinogenesis. REG Iα and REG IV are responsible for the tumorigenesis of colorectal (**A**) and gastric cancer (**B**).

As discussed above, REG protein is overexpressed in mucosal cells from *H. pylori*-infected subjects with concomitant chronic gastritis, which is a risk factor for the development and progression of gastric cancer [68]. Kadowaki et al. have reported that REG appears to be highly expressed in a large number of gastric cancers in vivo [69]. Reg could dose-dependently stimulate thymidine incorporation in MKN45 and AGS gastric cancer cells, partly through activation of the extracellular signal-regulated kinase (ERK) 1/2 and MAPK pathways. Moreover, Fukui et al. have demonstrated the expression of both *REG Iα* and *REG receptor* mRNA in seven human gastric cancer cell lines [70]. REG Iα-positive early gastric cancer also exhibits a markedly higher proliferating cell nuclear antigen labeling index and more severe inflammatory cell infiltration in the vicinity of gastric mucosa [71]. Another study has suggested that the expression of REG Iα is positively associated with microvessel density in gastric cancer [72]. Administration of REG Iα enhanced the proliferation and survival of endothelial cells via the ERK and Akt signaling pathways. Collectively, the evidence suggests that REG Iα protein serves as a trophic, antiapoptotic and angiogenetic factor for gastric cancer. A summary of these contents is demonstrated in Figure 3.

## 6. Reg Family Proteins as Biological Markers of Inflammatory/Neoplastic Diseases

A proteomic analysis using mass spectrometry has suggested that Reg Iα might be a strong biomarker in patients with infliximab-treated RA [73] (Table 1). In patients with celiac disease, Reg Iα expression is increased in the targeted tissue and also in serum during damage/inflammation, whereas it is decreased after a gluten-free diet, suggesting its potential applicability for diagnosis and monitoring of the disease [74]. In posttraumatic sepsis, serum Reg Iα is related to the severity of inflammation and activates neutrophil granulocytes [75]. An investigation by Que et al. has found that plasma Reg Iα was significantly higher in patients with septic shock than in those with severe sepsis and that the plasma Reg Iα concentration was the only biomarker associated with in-hospital mortality [76]. On the other hand, it has been shown that the plasma Reg Iα level does not differ between children with systemic inflammatory response syndrome and those with septic conditions until signs of organ dysfunction are present [77]. Ferrara et al. have reported that Reg IIIα can be used as a plasma biomarker of GI graft-versus-host disease in combination with clinical stage and histologic grade to improve risk stratification of patients [22]. The biomarker potential of Reg Iα/Reg IIIα has also been validated for identifying septic complication/infection in patients following a variety of surgical procedures [78,79].

Yonemura et al. have indicated that *REG Iα* gene expression is closely linked to the infiltrative potential of gastric carcinoma and might function as a prognostic indicator for differentiated adenocarcinoma of the stomach [80]. REG Iα expression is also an independent predictor of unfavorable outcomes in gastric cancers, irrespective of intestinal mucin phenotype [81]. In patients with stage IV gastric cancer, REG Iα is a potent predictive marker of resistance to chemotherapeutic drugs [82], and furthermore, serum REG IV protein is a novel predictive marker of resistance to 5-FU-based chemotherapy in patients with gastric cancer [83]. Tao et al. have found that, in TNM stage I and II patients with gastric cancer, the proportion of serum samples positive for REG IV is significantly higher than that of samples positive for carcinoembryonic antigen or carbohydrate antigen 19-9 [84].

Zheng et al. have reported that both REG Iβ and REG IIIα show lower expression in CRCs with venous invasion, lymph node metastasis and deeper invasion, suggesting their possible use as prognostic indicators [85]. In patients with early CRCs without metastasis undergoing potentially curative surgery, the expression of REG I, both alone and in combination with REG IIIα, predicts an adverse survival outcome [86]. Advanced CRC with metastatic hepatic recurrence is reported to show REG IV staining more frequently than that without such metastasis [87]. Increased preoperative serum REG IV levels have been found solely in stage IV CRC patients with liver metastasis. In addition, it has been proven that REG IV acts as a crucial modulator of CRC cell sensitivity and susceptibility to radiotherapy [88,89].

**Table 1:** Reg family members as biomarkers for inflammatory/neoplastic diseases.

Reg Family	Diseases	Findings	References
REG Iα	RA	The efficacy of infliximab treatment	[73]
REG Iα	Celiac disease	For diagnosis and monitor	[74]
REG Iα	Posttraumatic sepsis	Related to the severity of inflammation	[75]
REG Iα	Septic shock	Increased and associated with in-hospital mortality	[76]
REG Iα	SIRS	Increased at the onset of organ dysfunction	[77]
REG Iα	GC	Relevant to infiltrating pattern and function as prognostic factor	[80]
REG Iα	GC	Independent predictor of poor outcomes	[81]
REG Iα	Stage IV GC	Potent indicator for resistance to chemotherapy	[82]
REG I/REG IIIα	Early CRC	Indicator of adverse effect on survival	[86]
REG Iα/REG IIIα	Abdominal/cardiac surgery	Identification of septic complications	[78,79]
REG Iβ/ REG IIIα	CRC	Predictor of favorable prognosis	[85]
REG IIIα	Gastrointestinal GVHD	Prediction of response to therapy and 1-year survival following HCT	[22]
REG IIIα	Mucosal enteropathies	Useful marker to distinguish mucosal enteropathies from IBS	[90]
REG IIIα	CD	Increased in active conditions	[91]
REG IV	GC	Predictor of resistance to 5-FU-based chemotherapy	[83]
REG IV	GC	Prognostic indicator and early diagnosis of GC	[84]
REG IV	Advanced CRC	Prognosticator of poor survival with liver metastasis	[87]
REG IV	CRC	Crucial modulator of sensitivity and specificity to radiotherapy	[88,89]
REG IV	UC	Upregulation in remitting mucosa	[92]

RA, rheumatoid arthritis; SIRS, systemic inflammatory response syndrome; GVHD, graft-versus-host disease; HCT, hematopoietic cell transplantation; GC, gastric cancer; CRC, colorectal cancer; CD, Crohn’s disease; UC, ulcerative colitis; IBS, irritable bowel syndrome.

Marafini et al. have reported that serum REG IIIα assay is useful for distinguishing between mucosal enteropathies and functional intestinal conditions [90]. Moreover, serum REG IIIα levels decrease significantly following successful treatment with infliximab, supporting the feasibility of REG IIIα as an indicator of therapeutic efficacy. However, Nunes et al. have concluded that serum REG IIIα is upregulated in patients with active CD relative to those in remission, although its predictive capacity is relatively low, with an AUC of 0.69 [91]. Another study has shown that the expression of both *REG IV* mRNA and protein is markedly upregulated in the colonic mucosa of UC patients in remission, suggesting that further investigation of its possible role as a predictor of progression to CRC might be warranted [92].

## 7. Conclusions

Reg family proteins have versatile roles as trophic, antiapoptotic, angiogenetic, anti-inflammatory and bactericidal secretory molecules. The data available so far indicate that members of the Reg protein family are intimately involved in the occurrence, progression and development of many inflammation-associated GI tract conditions. It is anticipated that further research will lead to the adoption of these proteins and genes as therapeutic targets and prognostic biomarkers.

## Data Availability

Not applicable.

## Abbreviations

REG	regenerating gene in human
Reg	regenerating gene in mouse/rat
GI	gastrointestinal
IBD	inflammatory bowel disease
RA	rheumatoid arthritis
ECL	enterochromaffin-like
NF-κB	nuclear factor kappa B
TNF-α	tumor necrosis factor-α
UC	ulcerative colitis
EXTL3	exostoses-like gene 3
ATF-2	activating transcription factor-2
CRC	colorectal cancer
GPR37	G protein-coupled receptor 37
STAT3	signal transducer and activator of transcription 3
CDX2	caudal type homeobox 2
CD	Crohn’s disease
Th2	T-helper type 2
DSS	dextran sulfate sodium
GATA6	GATA DNA-binding protein 6
ERK	extracellular signal-regulated kinase
MAPK	mitogen-activated protein kinase
MZF1	myeloid zinc finger 1
RTEF1	related transcriptional enhancer factor-1
HLTF	helicase-like transcription factor
bFGF	basic fibroblast growth factor
HGF	hepatocyte growth factor
AP1	activator protein-1
EGFR	epidermal growth factor receptor
GSK-3β	glycogen synthase kinase 3β
TCF4	T-cell factor 4
SIRS	systemic inflammatory response syndrome
GVHD	graft-versus-host disease
HCT	hematopoietic cell transplantation
GC	gastric cancer
IBS	irritable bowel syndrome
